# The Effects of Topographical Patterns and Sizes on Neural Stem Cell Behavior

**DOI:** 10.1371/journal.pone.0059022

**Published:** 2013-03-18

**Authors:** Lin Qi, Ning Li, Rong Huang, Qin Song, Long Wang, Qi Zhang, Ruigong Su, Tao Kong, Mingliang Tang, Guosheng Cheng

**Affiliations:** 1 Department of Nanobiomedicine, Suzhou Institute of Nano-Tech and Nano-Bionics, Chinese Academy of Sciences, Suzhou, Jiangsu, P.R. China; 2 Graduate University of the Chinese Academy of Sciences, Beijing, P.R. China; The Ohio State University, United States of America

## Abstract

Engineered topographical manipulation, a paralleling approach with conventional biochemical cues, has recently attracted the growing interests in utilizations to control stem cell fate. In this study, effects of topological parameters, pattern and size are emphasized on the proliferation and differentiation of adult neural stem cells (ANSCs). We fabricate micro-scale topographical Si wafers with two different feature sizes. These topographical patterns present linear micro-pattern (LMP), circular micro-pattern (CMP) and dot micro-pattern (DMP). The results show that the three topography substrates are suitable for ANSC growth, while they all depress ANSC proliferation when compared to non-patterned substrates (control). Meanwhile, LMP and CMP with two feature sizes can both significantly enhance ANSC differentiation to neurons compared to control. The smaller the feature size is, the better upregulation applies to ANSC for the differentiated neurons. The underlying mechanisms of topography-enhanced neuronal differentiation are further revealed by directing suppression of mitogen-activated protein kinase/extracellular signaling-regulated kinase (MAPK/Erk) signaling pathway in ANSC using U0126, known to inhibit the activation of Erk. The statistical results suggest MAPK/Erk pathway is partially involved in topography-induced differentiation. These observations provide a better understanding on the different roles of topographical cues on stem cell behavior, especially on the selective differentiation, and facilitate to advance the field of stem cell therapy.

## Introduction

Neural stem cells (NSCs) are intrinsically capable of differentiating into different neural cell types: neurons, oligodendrocytes and astrocytes [1], which can supply new cells for treating neurodegenerative diseases and neurological injuries. However, the major obstacle for clinical NSC therapy is the lack of efficient methodologies for large-scale expansion and controlled differentiation to functional cell types for transplantation, thus enhanced control of NSC differentiation to special lineage cells becomes one of the critical issues for the success of NSC-based therapies [2], [3]. Although successful biochemical manipulation of NSC differentiation in vitro has been achieved through supplementation of various growth factors to the culture medium [4], [5], [6], how physical cues including topographical patterns and feature sizes, exert regulatory influence on NSC proliferation and differentiation remains to be elucidated.

The arising cognition on topographical cues controlling stem cell fate origins from the understanding of the regulatory role of naturally occurring topographic structures in cell structure and function. Basal lamina membranes in some tissues has unique nanofibrous characteristics [7], suggesting the functionality and importance of substrate topography, which can be verified by a work that cells could respond to topography environment in vivo [8]. Meanwhile, synthetic topography has further been proved to be capable of inducing different effects on cells, such as cell morphology, alignment, adhesion, migration, proliferation, and cytoskeleton organization [9]. Specially, more and more reports demonstrated that artificial biomaterials presenting topographical features such as pillars, grooves, or pits could affect the structure, proliferation and differentiation of various stem cells [9], [10]. Micro- and nano-topography generated by colloidal lithography, electron beam lithography and polymer demixing techniques have been shown to promote the osteogenic differentiation of human bone marrow-derived osteoprogenitors [11], [12] and human mesenchymal stem cells (hMSCs) [11]. Similarly, osteoblast differentiation has been observed in preosteoblast cells cultured on a nanofibrous poly (L-lactic acid) mesh prepared using phase separation method [13].

To date, which features of topographical cues, including pattern and size, play the dominant role in regulating NSC differentiation are not well understood. Considering that neural cells are one of the most notable examples of highly polarized cell types, anisotropic topographical cues should regulate cell behavior better than isotropic topographical cues, which can be supported by a recent study that microgrooved surfaces promoted neurite growth [14]. Here, we fabricated three anisotropic topographical cues (linear micropatterns: LMP, circular micropatterns: CMP and dot micropatterns: DMP) representing different patterns with various feature sizes (2 or 10 µm width and spacing) on Si wafers to elucidate the role of pattern and size on topography-induced biological effects on NSC. Using multipotent adult neural stem cells (ANSCs) as a model cell line, we assessed the potential of topographical cues to regulate ANSC morphology, survival and proliferation. Most importantly, different roles of topographical patterns and sizes on differentiation preference were explored. In addition, we investigated the topography-induced differential signaling activation of the MAPK/Erk pathway to address the proper functionality of the differentiated neurons from ANSCs.

## Materials and Methods

### Preparation and analysis of topography substrates

Fabrication of the LMP, CMP and DMP followed a standard photolithography process to obtain the topography substrate with average feature sizes of 2 µm and 10 µm diameter and spacing with 4 µm depth. All the substrates are ∼1 cm^2^ for cell culture except for western blot experiments, where larger (∼4 cm^2^) substrates were used. Firstly, AZ 5214 (AZ Electronic Materials, USA) photoresist was spin-coated on pre-cleaned Si wafers with a coating rate of 4000 rpm for 30 s, followed by 95 °C prebake for 1 min. MA6/BA6 aligner (Suss Microtech, Germany) was then utilized to expose the patterns of different topographies under vacuum mode. The exposure time was set to be 7 s to obtain the designed feature sizes. After development, the wafers with photoresist patterns were then etched to acquire 4 µm depth in an ICP etching system (STS MPX HRM) by sophisticatedly adjusting the etching parameters (45 W, SF_6_: 450 sccm, O_2_: 45 sccm, C_4_F_8_:100 sccm, 45 s). After removal of the photoresist by acetone and isopropyl alcohol, the wafers were ready for further characterizations and experiments. In all experiments, the flat and blank Si without topography was used as control substrate.

The surface topographies of the wafers were observed by SEM (Inspect S, FEI, USA). The surface chemistry of the substrates pre- and post- polyornithine (PLO) and laminin (LN) modification was examined using XPS (Axis Ultra DLD, Kratos, UK) utilizing an Al Kα X-ray source operated at 40 eV.

### ANSCs culture in proliferation and differentiation conditions

ANSCs were derived from both hemispheres hippocampus of postnatal day 1 ICR rat (Animal Center in SooChow University). Hippocampus was removed from blood vessels and meninges to be collected in falcon tubes in hank's balanced salt solution (HBSS) at 4 °C, then rinsed with HBSS solutions for two times. After centrifugation (1000 r/min for 5 mins), tissues were digested in TryplE (Life Technologies, USA) for 15 min at 37 °C, then gently triturated mechanically by using pipet tips to ANSC suspension in DMEM-F12 medium containing 2% B-27.

Before cell seeding, all culturing substrates were sequentially coated with PLO (10 mg/ml, 37°C for 2 h, Sigma, USA) and laminin (20 mg/ml, 37°C overnight, Sigma, USA). ANSCs were cultured at 37°C in humidified atmosphere with 5% CO_2_. Passage of ANSCs was carried out every 7-day culturing. For proliferation studies, ANSCs were seeded at a concentration of 5×10^4^ cells/mL in proliferation condition of DMEM-F12 medium containing 2% B-27 by supplementation of 20 ng/mL EGF and 20 ng/mL FGF-2(R&D Systems, USA). For differentiation studies, ANSCs were cultured at the similar concentrations. ANSCs were firstly seeded in the DMEM-F12 medium containing 2% B-27 by supplementation of fetal bovine serum (FBS, GIBCO, USA) and 1 µM RA (Sigma, USA) and can be differentiated into a mixed lineage of neurons, astrocytes and oligodendrocytes. ANSCs in proliferation and differentiation conditions were cultured for 7 days before imaging and immunocytochemical analysis.

The care and use of animals in these experiments followed the guidelines and protocol approved by the Care and Use of Animals Committee of Suzhou Institute of Nano-Tech and Nano-Bionics. All efforts were made to minimize the number of animals used and their suffering.

### Assessment of ANSC viability

To evaluate the viability of the ANSCs seeded onto topography substrates, the substrates with ANSC (a concentration of 5×10^4^ cells/mL) were rinsed with phosphate buffered saline (PBS) for two times after 7 days of culture in proliferation condition. The topography substrates were incubated in PBS containing 2×10^−4^ mM of calcein-AM and 2×10^−4^ mM of EthD-1 for 40 min at 37°C. Then the substrates with ANSCs were rinsed three times (5 min for each). The live ANSCs stained with calcein-AM show green color and the dead stained with EthD-1 show red color under a Nikon Ti-E fluorescent microscope. ANSCs survival rate was calculated by determining the percentage of calcein-AM-positive cells over total cell number, and ANSCs death rate was calculated by determining the percentage of EthD-1-positive cells over total cell number. For each experimental group, at least ten fields of each substrate (including 4 corners and one centre) were imaged.

### Immunofluorescence staining and imaging of ANSCs

Immunofluorescence staining was performed to characterize phenotypic changes of ANSCs that occurred over the course of proliferation or differentiation culture. Cells were washed with PBS two times (3 min for each), fixed in 4% paraformaldehyde at room temperature for 40 min, blocked with 0.25% Triton X-100 (Beyotime, China) overnight. Primary antibodies were incubated for 3 hours, and secondary antibodies were incubated for 1.5 hours, followed by DAPI (nuclear marker, Molecular Probes, USA) staining for 15 min at room temperature. Antibody panel used include primary antibody against nestin (neural progenitor marker, mouse, Abcam, 1∶200, Abcam, USA), Tuj-1 (neuronal marker, rabbit, 1∶1000, Sigma), RIP (oligodendrocyte marker, mouse, Santa Cruz Biotechnology,USA), O4 (oligodendrocyte marker, rabbit, 1∶1000, Abcam), GFAP (astrocyte marker, rabbit, 1∶1000, Abcam) and β-tubulin (cytoskeleton marker, mouse, 1∶1000, Sigma), which were used for differentiation conditions; Ki-67 (rabbit, 1∶500, Abcam) was additionally used for proliferative investigation. Afterwards, ANSCs were rinsed with PBS and incubated with fluorescently labeled secondary antibodies Fluor®488-conjugated (1∶200) and Fluor ®568-conjugated (1∶200) (Invitrogen, USA) in PBS for 60 min at room temperature.

Three samples of each substrate type were imaged, with overlapping staining combinations to minimize non-specific quantification using the fluorescence microscope. At least 20 images of each staining condition were captured at 200× magnification and manually counted to obtain positive stained fractions (DAPI staining was used as counter staining). Photographs of labeled proteins were taken from fiuorescence microscopy and analyzed using NIS-Elements Viewer 3.20. The final results were derived from the average of three sets of experiments.

### Western blot analysis

Western blot was used to analyze the differentiation situations of ANSCs with or without ERK signaling pathway inhibitor U0126 (Cell Signaling, USA) on topography substrates. ANSCs dissociated from neurospheres were seeded on various substrates in differentiation condition. When ERK signaling pathway inhibitor was used, ANSCs (a concentration of 5×10^4^ cells/mL) were pre-incubated with U0126 for 1 h prior to medium. About 50 µg of protein was loaded onto SDS-polyacrylamide gels and underwent electrophoresis at 90 V for 30 min. The separated protein was then transferred to a nitrocellulose membrane at 110 V for 70 hour in a Tris-glycine transfer buffer (Invitrogen). The membrane were then blocked in 5% nonfat dry milk for 1 h and incubated overnight at 4°C with anti-Nestin, Tuj-1, RIP, O4 and GFAP-antibodies. Horseradish peroxidase conjugated goat anti-mouse or anti-rabbit IgG (ECL Kit; Amersham, Arlington Heights) was then applied for 1 h at room temperature. The blots were developed in luminal and exposed to Hyperfilm ECL (Amersham). The blots were stripped and normalized by re-probing with a gel loading (β-tubulin or β-actin) control. The molecular weights were compared with prestained low-range standards (Bio-Rad, USA).

### Statistical analysis

All data were presented as the mean ± standard error of the mean (SEM) of at least 3 independent experiments. Statistical analysis was performed using the two-way analysis of variance (ANOVA) test followed by a Tukey test (LST) to evaluate the statistical significance between the different groups. The significance levels were set at *p<0.05 and **p<0.01.

## Results

### Characterization of substrate topography

**Figure 1:**
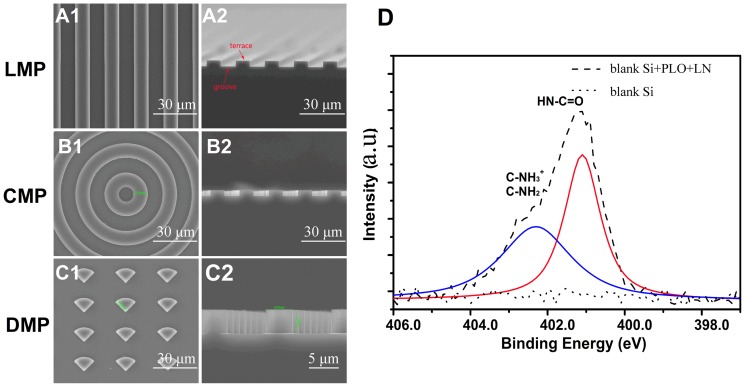
Representative SEM images of LMP (A1), CMP (B1), DMP (C1) substrates and their corresponding cross-section images (A2, B2, C2). (D) High-resolution XPS spectra of blank silicon substrates before (dot) and after (dash) PLO-LN coating. Scale bar  = 30 µm (A1–C1) and 5 µm (C2).

Standard photolithography process was employed to fabricate silicon topological substrates. Surfaces of various topological substrates were observed using SEM (**Figure 1A–C**). The topography substrates used in this study were in around 10 µm width, 10 µm spacing and 4 µm depth, except for the experiments of ANSC differentiation in which additional patterns with 2 µm width, 2 µm spacing and 4 µm depth were used. The average area of substrates for cell culturing was approximately 1 cm^2^. Besides, all culturing substrates were pre-coated with laminin (LN, 20 mg/ml, 37°C, overnight, Sigma, USA), which is believed to be useful for NSC growth. Surface chemistry of silicon substrates coated with or without LN was examined by X-ray photoelectron spectroscopy (XPS) (**Figure 1D**). We can quantify LN coverage by measuring N content as N contained in the silicon forming the LN molecule, proving the success of LN modification, which was further validated by presence of the N 1s peaks (HN-C = O, C-NH_2_, C-NH_3_^+^) (**Figure 1D**).

### ANSCs morphology on different substrates

**Figure 2:**
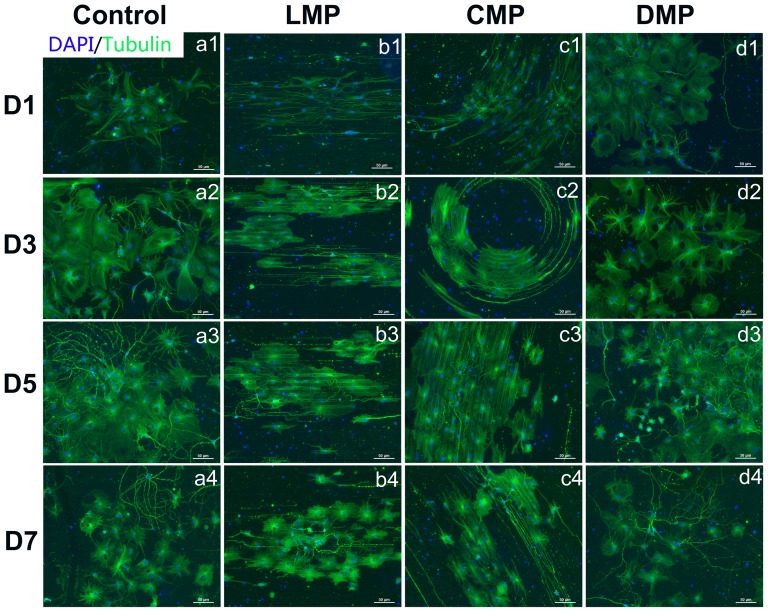
Representative fluorescent images of ANSCs under differentiation conditions on control (a1–a4), LMP (b1–b4), CMP (c1–c4) and DMP substrates (d1–d4). Cell nuclei were labeled with DAPI (blue) and cells were immunostained with anti-β-tubulin antibody (green). ANSCs were prepared for the labeling after 1 day (a1, b1, c1, d1), 3 days (a2, b2, c2, d2), 5 days (a3, b3, c3, d3) and 7 days (a4, b4, c4, d4) of differentiation, respectively. Images were acquired at 200× magnification and the scale bar  = 50 µm.

Many cell types could respond to topography patterns, especially micro- and nano-grooves or gratings, by simultaneously aligning and elongating the cell bodies and neuritis in the direction of the grating axisMany cell types could respond to topography patterns, especially micro- and nano-grooves or gratings, by simultaneously aligning and elongating the cell bodies and neuritis in the direction of the grating axis [9]. To detect the ANSC morphologies, we investigated the morphological change of ANSCs cultured on topography substrates in serum-free medium for 1, 3, 5 and 7 days, respectively. Cells were stained with β-tubulin for cell cytoskeleton (green in **Figure 2**) and DAPI for cell nucleus (blue in **Figure 2**). ANSCs could adhere to all substrate surfaces on the first day after seeding and show similar density. Cell bodies and extending branches of ANSCs exhibited random distribution and showed no preference of direction on the control (**Figure 2a** **1–a4**) and DMP substrates (**Figure 2d** **1–d4**), while cell nucleus elongated along the axis of the grooves and cell extended branches that were guided by topological directionality on LMP (**Figure 2b** **1–b4**) and CMP (**Figure 2c** **1–c4**). This phenomenon existed within the whole culturing time.

### Cell viability on substrate topography

**Figure 3:**
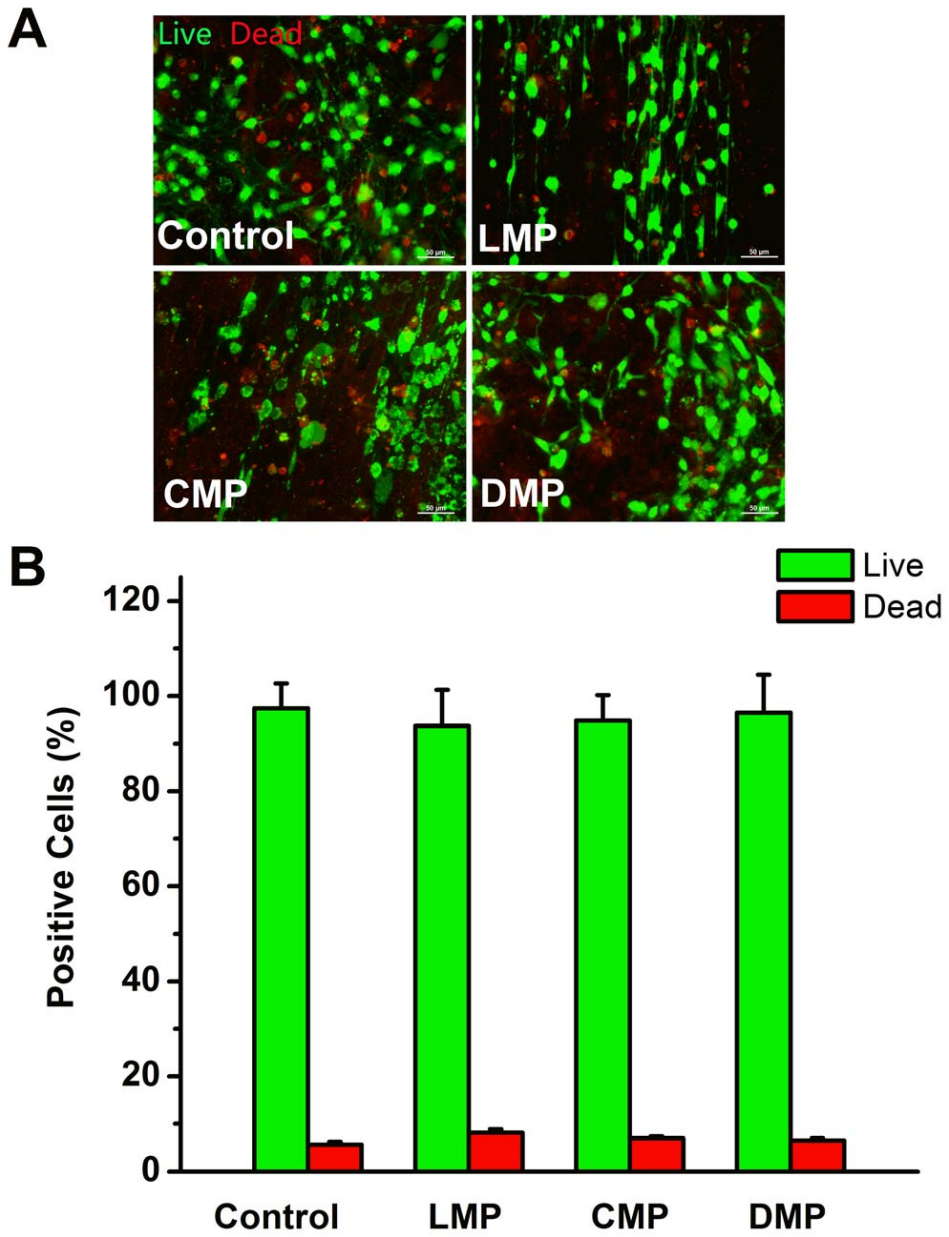
Fluorescence microscopic images of the ANSCs on control, LMP, CMP and DMP substrates. (A) Cells were stained by calcein-AM and EthD-1 staining. Viable cells were labeled by the green fluorescence generated from the esterase hydrolysis of a membrane-permeant dye, calcein-AM. Dead cells were marked by the red fluorescence of a membrane-impermeant DNA marker, EthD-I. (B) The statistic data of percentages of live and dead cells in control, LMP, CMP and DMP groups. Scale bar = 50 µm.

To examine cell viability on these substrates, ANSCs were cultured for 7 days in differentiation condition, and stained with calcein-AM and ethidium homodimer-1 (EthD-1). Viable cells exhibited green fluorescence that was generated by the esterase hydrolysis of membrane-permeant dye, calcein-AM. Dead cells were marked by red fluorescence EthD-1 (**Figure 3A**). More than 400 cells were calculated for each group. The percentages of viable and dead cell were 97.4±5.2% and 5.7±0.6%, 93.7±7.5% and 8.1±0.8%, 94.8±5.4% and 6.9±0.4%, and 96.5±7.9% and 6.5±0.5% on control, LMP, CMP and DMP, respectively (**Figure 3B**). There were no significant differences on cell viability between each group (n = 3, *p*>0.05).

### Effect of substrate topography on ANSC proliferation

**Figure 4:**
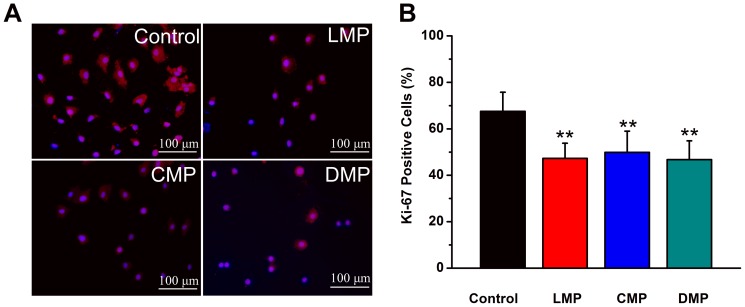
Effect of substrates topography on ANSCs proliferation in serum free medium for 7 days. (A) Shown are representative images of Ki-67 antibody-stained cells (red) and DPAI-stained nuclei (blue) cultured on control, LMP, CMP and DMP substrates. Scale bar = 50 µm. Quantitative analysis of Ki-67 positive cells is shown in (B). Bars represent mean ± SEM (n = 3); ** indicates statistical significance (*p*<0.01) compared with control group. Scale bar = 50 µm.

In order to characterize the effect of substrate topography on ANSC proliferation, we expanded ANSCs in serum free medium in the presence of EGF and FGF-2, two sufficient mitogens for NSC proliferation. ANSC proliferation was characterized by positive Ki-67 staining after 7 days of culture (**Figure 4A**) and around 300 cells were counted for each group. Among all substrates tested, control substrate was most effective to promote ANSC proliferation to the ratio of 67.5±8.2%. While on LMP, CMP and DMP substrates, proliferation rates were significantly decreased to 47.2±6.5%, 49.9±9.1% and 46.7±8.1% (**Figure 4B**, n = 3, *p*<0.01 compared to control), respectively.

### Differentiation of ANSCs on substrate topography

**Figure 5:**
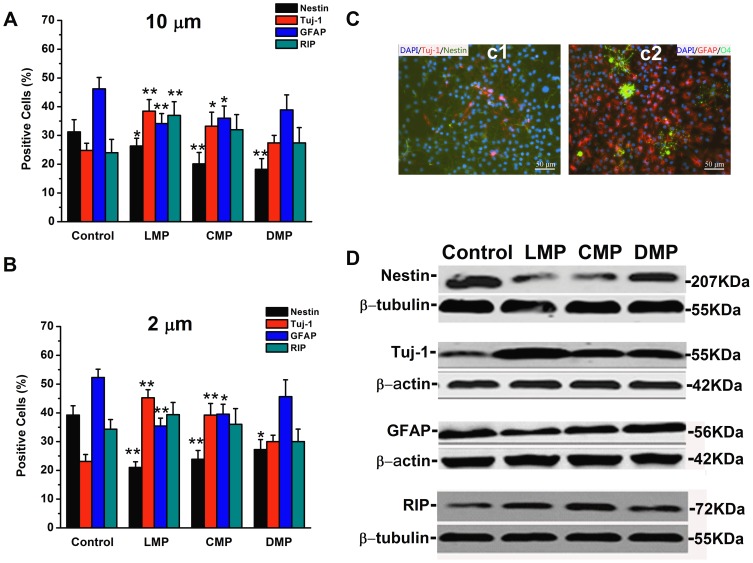
Immunofluorescence analysis of ANSCs cultured on various substrates in the presence of 1 µµM retinoic acid and 2% fetal bovine serum for 7 days. Cell nuclei (blue) were stained using DAPI as a counter staining. Quantification of staining results is shown on (A) 10 µm width substrates of each pattern and (B) 2 µm width substrates of each pattern. Error bars represent mean ± SEM (n = 3, more than 400 cells were counted for each sample). (C) Representative fluorescent images of cells stained positive for (c1) Tuj-1 and nestin and positive for (c2) GFAP and O4. (D) Western blot analysis of nestin, Tuj-1, GFAP and RIP expression in ANSCs on each substrate under differentiation conditions for 7 days. (*p<0.05 and **p<0.01). Scale bar = 50 µm (C).

To determine whether ANSCs were enhanced to differentiate by substrate topography, ANSCs were cultured for 7 days in the presence of 1 µM retinoic acid (RA) and 2% fetal bovine serum (FBS). Cells were stained with different cell type markers and immunofluorescence results showed ANSCs differentiated into all three major cell types (**Figure 5C**): neurons, astrocytes, and oligodendrocytes. For immunofluorescent statistics, over 300 cells were observed for each group. For substrate topography in 10 µm width wafers (**Figure 5A**), the percentage of stained positive cells for nestin was 31.2±4.3% on control, and it was significantly decreased to 26.3±2.7% (n = 3, *p*<0.05), 20.1±3.9% (n = 3, *p*<0.01) and 18.2±3.7% (n = 3, *p*<0.01) on LMP, CMP and DMP substrates, respectively. Meanwhile, ANSCs showed a significantly increase by 13.67% in neuronal differentiation on LMP substrates (38.5±4.0%) compared to on control (24.8±2.5%, n = 3, *p*<0.01), while a smaller increase by 8.42% on CMP substrates (33.2±4.9%, n = 3, *p*<0.05) and a slight increase by 2.6% on DMP substrates (27.4±2.6, n = 3, *p*>0.05). On the contrast, ANSCs differentiation to astrocytes was significantly decreased by 12.1% and 10.2% on LMP (34.1±3.5%, n = 3, *p*<0.01) and CMP substrates (36.0±4.2%, n = 3, *p*<0.05), respectively, when compared to control (46.2±3.9%, n = 3), while there was no obvious difference in astrocyte differentiation on DMP (38.9±5.2%, n = 3, *p*>0.05 compared to control). ANSCs showed increased differentiation to oligodendrocyte by 12.9%, 8.0% and 3.4% on LMP (36.9±4.8%, n = 3, *p*<0.01), CMP (32.0±5.2%, n = 3, *p*>0.05) and DMP substrates (27.4±5.3%, n = 3, *p*>0.05) respectively, compared to control (24.0±4.6%). Similar results can be seen in substrates topography groups in 2 µm width (**Figure 5B**), while LMP and CMP both promoted ANSCs differentiation into neurons to a greater extent compared to that of corresponding patterns in 10 µm width, with the ratio of 45.2±2.8% and 39.2±4.0%, respectively. Western blot analysis of substrate topography in 10 µm width showed similar results with the immunofluorescent statistical data (**Figure 5D**). All three topographical substrates could downregulate nestin expression compared to control, while all upregulate tuj-1 expression, especially on LMP substrates.

### The Effects of MAPK/ERK signaling pathway on topography-enhanced-differentiation

**Figure 6:**
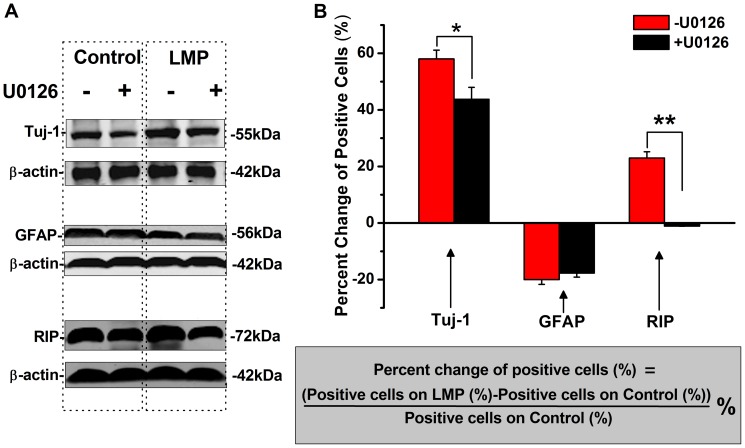
MAPK/Erk signaling pathway was involved in topography-enhanced preferential differentiation. (A) Western blot results of Tuj-1, GFAP and RIP protein expression on control and LMP substrates either in the presence or absence of U0126. (B) Column figure for percent change of Tuj-1, GFAP and RIP positive cells before and after U0126 treatment in the immunostaining experiments (n = 3 for each sample). The formula in the gray panel represents the method of calculating the percent change. *p<0.05, **p<0.01.

The mechanisms that underlying topography enhanced neuronal differentiation of ANSCs were further investigated in LMP group using U0126, a classic inhibitor of ERK signaling pathway which involves the regulation of neuronal differentiation in NSCs. To quantify the differentiation variation on LMP substrates, corresponding changes in Tuj-1, GFAP and RIP immunofluorescent positive cells were calculated compared to that of control with or without U0126 treatment. As shown in **Figure 6B**, U0126 treatment could partially depress the percent change of Tuj-1 positive cells from 58.0±3.1% to 43.7±4.2% (n = 3, *p*<0.05), and even reverse the percent change of RIP positive cells from 23.0±2.2% to −1.1±0.1% (n = 3, *p*<0.01). While it had no significant impact on the percent change of GFAP positive cells (−20.0±1.7% to −17.7±1.4%, n = 3, *p*>0.05). The percent changes of protein expression of Tuj-1, GFAP, and RIP analyzed by western blot show consistent results (**Figure 6A**), indicating ERK signaling pathway was involved in topography-enhanced-differentiation of ANSCs.

## Discussion

It is critical to increase cellular alignment and axonal guidance for neural regeneration and tissue engineering. In this study, the strategy to choose anisotropic topographical patterns (LMP, CMP and DMP), rather than isotropic ones, is mainly based on the common knowledge of that the neurons are highly polarized cell types. Engineered anisotropic topographical cues, i.e. micro-grooves and aligned nanofibers, could improve neurite outgrowth and promote functional connections [14], [15]. Also, anisotropic patterns, especially those with obvious directions (LMP and CMP in our study), resemble the extracellular matrix in the physiological condition. Meanwhile, Si wafers are chemically stable and industrially established for fabrication to well-defined topographical substrates, which is ideal for studying the real effects of topography on cells. All tested Si substrates were pre-treated with LN before ANSCs were seeded in the experiments. LN is a key component of the basement membrane which could mediate NSC adhesion as the most efficient cell adhesion molecule [16] and enhance cellular response to topography [17], [18]. XPS data show successful modification of the substrates with LN (**Figure 1B**). NSCs could adhere to the substrates pretty well and show normal viability compared with those on the control after coating with LN (**Figure 3**).In contrast, ANSCs were unable to adhere on the Si substrates and failed to exhibit the capability of differentiation without LN coating even in the presence of serum (data not shown).

It is reported that the most palpable effect of topography on cell function is due to the impact upon cell geometry [9]. Our results show that cell bodies and branches on the LMP and CMP with both 2 and 10 µm width substrates aligned and elongated in the direction of the grating axis (**Figure 2**), which is consistent with many reports that the alignment and extension of various cell types, including fibroblasts, endothelial cells, stem cells and Schwann cells, could be simultaneously directed by grating axis [19]. There is no obvious preference of the direction of alignment and extension for those cells cultured on the DMP with both 2 and 10 µm width and control substrates (**Figure 2**). Interestingly, other studies report that substrates with nanopost and nanopit features elicit a more subtle effect on cellular morphology [9], which is quite similar with ANSCs spreading and morphology on DMP substrates in our study. Meanwhile, previous studies found topography is able to affect the proliferation profiles of human embryonic stem cells [20] and human mesenchymal stem cells (hMSCs) [21]. Our results also show ANSCs cultured on all three topographical substrates exhibit lower proliferation rates than that on control (**Figure 4**). Recent studies reveal that micro- or nano-gratings could inhibit cell proliferation [20], [21], while the effect of other micro- or nano-patterns as nanopost or nanopit have different effects on proliferation [12], [22], [23]. To date, there are no widely accepted hypotheses regarding the mechanism for the effects of topography substrates on cell proliferation. The lower proliferation rate of ANSCs on the patterned substrates in our study may be due to the enhancement of ANSC differentiation either to neurons or glia cells.

There has been significant progress in the study of topographical cues potentially being utilized as a signaling modality for directing differentiation despite the fact that coordinated work has only recently been explored. However, it would be difficult to conclude which features (pattern or size) of topographical cue determining differentiation direction among current literatures. Disparity in material features, variation in topographical fabrication and different choosing of patterns (gratings, pit and post etc.) and sizes (from nanometer to micrometer) sometimes produce conflicting results. For example, the stiffness of the substrates may influence the differentiation, where softer substrates could favor neural differentiation [24]. It is consistent with our study, since the differentiation rates of cells cultured on Si wafers are higher than that on TCPS and PDMS substrates (data not shown). In this study, we try to address the roles of topographical patterns and feature sizes to govern NSC differentiation. The differentiation situations on the three patterns with 2 or 10 µm width were examined when ANSCs were cultured in the presence of 2% FBS and 1 µM RA. The results suggest different topographical patterns could induce ANSC differentiation to specific lineages under both feature sizes, where LMP and CMP were capable of depressing ANSC differentiation to astrocytes, instead of boosting ANSC differentiation to neurons (**Figure 5**). Especially, with smaller feature size of 2 µm width compared to 10 µm, both of LMP and CMP could induce neuronal differentiation to a greater extent (**Figure 5A and 5B**). Now, it is safe to draw a conclusion that the topographical patterns determine NSC differentiation fate and their feature sizes dictate the extent of differentiation preference. The differentiation situations of a variety of stem cells including embryonic stem cells, MSCs, hematopoietic stem cells, and NSC have been studied on different patterns of topographical surfaces. For example, Park *et al.* found that TiO_2_ nanotube layers with 15**–**30 nm diameters could support MSCs differentiation into osteogenic lineages at a higher rate than on polished TiO_2_ surfaces [25]. Similarly, another group reported that neuronal differentiation in hMSCs could be achieved on PDMS micro- and nanogratings [21]. More recently, fiber topography of electrospun substrates was proved to play a vital role in regulating differentiation and proliferation of rNSCs in vitro [26]. All these works indicate that substrates topography with certain patterns may induce stem cell differentiation to a specific lineage. These studies above exactly support our finding that topographical pattern could be used to determine stem cell fate. In the current literatures, different micro- and nano-scopic topologies such as grafts, posts, grooves and pits were used to attempt to induce preferred differentiation. Some studies suggest that micro- and nano-gratings and grooves could induce neuronal differentiation better than posts and pits [27]. Similarly, neuronal differentiation rates on LMP and CMP substrates are higher than that on DMP, which should be due to the nature of polarity of neural cells and the comparative anisotropic structure of LMP and CMP. The next question is what role of another important character of topographical cue, feature size. Two categories of feature sizes (2 µm or 10 µm in width and spacing, respectively, both 4 µm in depth) were employed to make sure that the cells will not be confined in the grooves in accordance to the soma diameter (usually ∼13 µm). Several studies revealed that differentiation rates of stem cells on micro-patterned substrates were affected by the comparison of sizes between patterns width and the soma diameter [14], [28]. It was reported that differentiation rate was affected for micropatterns smaller than the soma diameter, while there was no comparable differences if larger than the soma diameter[14]. Also, Recknor *et al.* did not observe any differences of selective differentiation of neural progenitor cells on micro-channels larger than the soma diameter as compared to a flat surface [28]. Work by Yim *et al.* suggests that hMSCs cultured on micro- and nano-gratings with various width of 350 nm, 1 µm and 10 µm could preferentially differentiated into neuronal lineages, while smaller width could bring bigger effects [21], which is quite consistent with our results. Indeed, neuronal differentiation rate of stem cells cultured on micro-patterned substrates is believed to be related to patterns width... Here, although the patterns widths (10 and 2 µm) are both smaller than the soma diameter, ANSCs still show different extents when differentiated into neuronal lineage on the two sizes of substrates. In conclusion, feature sizes may not be able to direct NSC differentiation preference, but proper sizes could boost controlled differentiation induced by topography to a maximal extent.

For now, the detailed mechanistic understanding of the relationship between micro- and nano-scale surfacing and the preference of stem cell differentiation is still lacking. Nevertheless, most of the researchers agree that cell-topography interactions should include the initial effects on cell adhesion and spreading, following subsequent gene expression, proliferation and differentiation [9]. Although the molecular mechanism for topographical sensing by cells remains largely undetermined, existing evidence from various cells suggests the involvement of integrin-mediated focal adhesion signaling [29]. Local micro- and nano-topographical cues can be sensed by adherent cells, thus integrins mediate cell adhesion to extracellular matrix and contribute to cell-matrix signaling by activating intracellular tyrosine kinase and phosphatase signaling, i.e., MAPK/Erk pathway, to elicit subsequent biochemical reactions, which may regulate stem cell fate [30], [31]. Naturally, we focus on the mechanism study of MAPK/Erk signaling pathway, which is involved in regulation of differentiation on NSCs into neurons or astrocytes [32] and another group reports that preferred differentiation in NSCs on ultra-nanocrystalline diamond films is mediated through the activation of MAPK/Erk kinases [31]. The percent change in specific lineage (neurons, astrocytes, oligodendrocytes) under U0126 treatment shows that MAPK/Erk signaling pathway was involved, at least partially, in topography-induced preferred differentiation in our study (**Figure 6**). Further study should validate whether the enhanced-differentiation by topography patterns is only through MAPK/Erk signaling pathway or not.

In summary, the present study demonstrated that all LMP, CMP and DMP topography substrates with LN modification did not influence ANSC survival, but depressed ANSC proliferation. LMP and CMP substrates with 10 and 2 µm width could promote the differentiation of ANSCs into neurons, while discourage the differentiation into astrocytes. The pattern of topographical cues could govern the preference of differentiation of NSCs, while the feature size could affect the extent of selective differentiation. The topography-induced preferential differentiation to neurons was found by the activation of MAPK/Erk signaling pathway. Based on the present study, future studies should be focused on screening the most effective topographical patterns and sizes to manipulate NSC differentiation and elucidating the detailed underlying mechanisms.
